# Early prediction of need for invasive mechanical ventilation in the neonatal intensive care unit using artificial intelligence and electronic health records: a clinical study

**DOI:** 10.1186/s12887-023-04350-1

**Published:** 2023-10-23

**Authors:** Younga Kim, Hyeongsub Kim, Jaewoo Choi, Kyungjae Cho, Dongjoon Yoo, Yeha Lee, Su Jeong Park, Mun Hui Jeong, Seong Hee Jeong, Kyung Hee Park, Shin-Yun Byun, Taehwa Kim, Sung-Ho Ahn, Woo Hyun Cho, Narae Lee

**Affiliations:** 1 Department of Pediatrics, Pusan National University School of Medicine, 20, Geumo-Ro, Mulgeum-Eup, Yangsan, 50612 Republic of Korea; 2 VUNO Inc, Seoul, Korea; 3 Division of Pulmonology, Allergy and Critical Care Medicine, Department of Internal Medicine, Pusan National University School of Medicine, and Research Institute for Convergence of Biomedical Science and Technology, Pusan National University Yangsan Hospital, Yangsan, Korea; 4 Department of Neurology, Division of Biostatistics, Research Institute for Convergence of Biomedical Science and Technology, Pusan National University Yangsan Hospital, Pusan National University School of Medicine, Busan, Korea

**Keywords:** Deep learning, Neonatal intensive care, Newborn, Respiratory failure, Intubation

## Abstract

**Background:**

Respiratory support is crucial for newborns with underdeveloped lung. The clinical outcomes of patients depend on the clinician’s ability to recognize the status underlying the presented symptoms and signs. With the increasing number of high-risk infants, artificial intelligence (AI) should be considered as a tool for personalized neonatal care. Continuous monitoring of vital signs is essential in cardiorespiratory care. In this study, we developed deep learning (DL) prediction models for rapid and accurate detection of mechanical ventilation requirements in neonates using electronic health records (EHR).

**Methods:**

We utilized data from the neonatal intensive care unit in a single center, collected between March 3, 2012, and March 4, 2022, including 1,394 patient records used for model development, consisting of 505 and 889 patients with and without invasive mechanical ventilation (IMV) support, respectively. The proposed model architecture includes feature embedding using feature-wise fully connected (FC) layers, followed by three bidirectional long short-term memory (LSTM) layers.

**Results:**

A mean gestational age (GA) was 36.61 ± 3.25 weeks, and the mean birth weight was 2,734.01 ± 784.98 g. The IMV group had lower GA, birth weight, and longer hospitalization duration than the non-IMV group (*P* < 0.05). Our proposed model, tested on a dataset from March 4, 2019, to March 4, 2022. The mean AUROC of our proposed model for IMV support prediction performance demonstrated 0.861 (95%CI, 0.853–0.869). It is superior to conventional approaches, such as newborn early warning score systems (NEWS), Random Forest, and eXtreme gradient boosting (XGBoost) with 0.611 (95%CI, 0.600–0.622), 0.837 (95%CI, 0.828–0.845), and 0.0.831 (95%CI, 0.821–0.845), respectively. The highest AUPRC value is shown in the proposed model at 0.327 (95%CI, 0.308–0.347). The proposed model performed more accurate predictions as gestational age decreased. Additionally, the model exhibited the lowest alarm rate while maintaining the same sensitivity level.

**Conclusion:**

Deep learning approaches can help accurately standardize the prediction of invasive mechanical ventilation for neonatal patients and facilitate advanced neonatal care. The results of predictive, recall, and alarm performances of the proposed model outperformed the other models.

**Supplementary Information:**

The online version contains supplementary material available at 10.1186/s12887-023-04350-1.

## Background

Adaptation to the extra-uterine environment is critical for the survival of neonates, and respiratory support is crucial in the neonatal intensive care unit (NICU). Particularly in preterm infants, lung immaturity can cause respiratory failure (RF) [1]. Every patient has various etiologies, symptoms, and progression of lung disease and different types of respiratory support devices used for treatment [2]. In the case of severe RF in neonates, invasive mechanical ventilation has been considered a life-saving treatment [1, 2]. The administration of therapies such as surfactant replacement or corticosteroids differs between NICUs depending on the physician’s experience [3]. Similarly, decisions regarding invasive mechanical ventilation (IMV) use also differ. The clinical outcomes of patients depend on the clinician’s ability to recognize the underlying status of the presented symptoms and signs. Multiple factors influence RF; therefore, accurately identifying neonates at risk for developing RF is a significant challenge for clinicians. Despite clinical advances, newborn morbidity and mortality remain high globally [4].

NICUs continuously monitor the physiological parameters of neonates, and physicians are confronted with plenty of data from many patients stored in electronic health records (EHR). Identifying the most important information required to make care decisions has become increasingly difficult. Furthermore, false-positive alarms can occasionally lead to alarm fatigue, negatively influencing clinicians [5]. The limited ability of humans to process such an enormous amount of data can lead to information overload. Thus, Artificial intelligence (AI) has begun to penetrate the healthcare systems in the NICU [6–10]. AI techniques have been developed over the past few decades [11]. These techniques range from traditional machine learning (ML) classifiers, such as eXtreme gradient boosting (XGBoost), Random Forest, support vector machine (SVM), and linear discriminant analysis (LDA), to deep learning (DL) models, such as artificial neural networks (ANN), convolutional neural networks (CNN), and long short-term models (LSTM) [12]. DL techniques help analyze complex signals with vast amounts of information [13]. Establishing high-quality, valuable, and multidimensional neonatal datasets can provide accurate prediction models. With the increasing number of high-risk infants, AI should be considered as a tool for personalized neonatal care; however, it is not widely used for newborns, and there are only a few DL studies related to neonatal lung disease [10]. Recent studies have investigated the potential of ML in predicting a wide range of neonatal outcomes, including sepsis, morbidity, retinopathy of preterm birth, and neural development [14–17].

This study sought to develop DL prediction models for the swift and precise detection of mechanical ventilation requirements in neonates using EHR. Moreover, our goal was to create a DL model that can be applied across all hospital tiers using data obtained non-invasively.

## Materials and methods

### Study design and participants

**Fig. 1:**
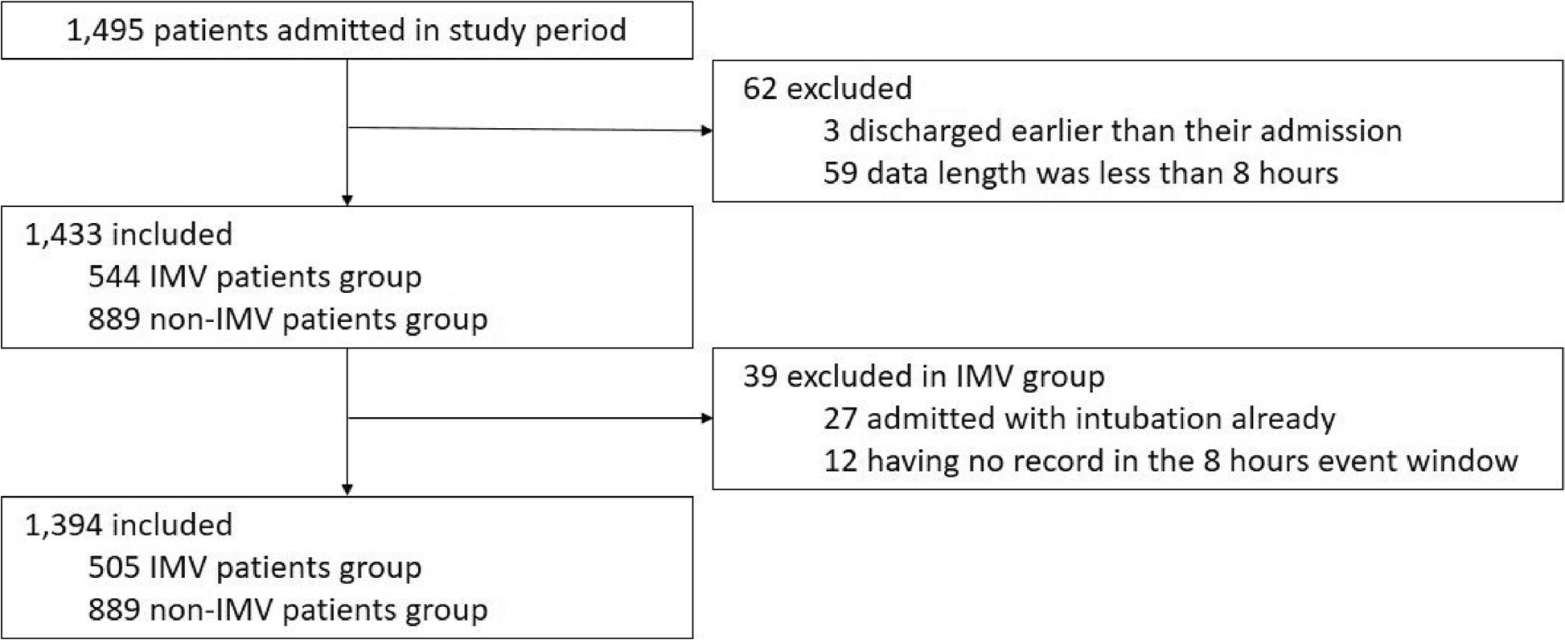
Flow chart of the study design

As shown in Fig. 1, the data used in this study were collected from the NICU of Pusan National University Yangsan Hospital in Korea from March 3, 2012, to March 4, 2022. During this period, data from 1,495 patient data were collected. Three patients discharged before admission and 59 patients with a data record of less than 8 h were excluded from the dataset. In addition, 27 patients who were intubated at admission and 12 patients who had no record for 8 h prior to its occurrence were excluded from the experiment because the model predicted the timing of intubation 8 h in advance. Finally, the data used for the model development consisted of 1,394 patients, including 505 patients in the IMV group and 889 patients who were not.

### Risk factor selection

The factors learned by this model are widely used risk factors important for predicting IMV support in neonates [1, 18, 19]. These factors are mainly composed of demographic characteristics and vital data, which can be obtained in a non-invasive manner and are essential for measurement in most NICUs from primary to tertiary hospitals, including gestational age (GA), birth weight, height, head and chest circumference, sex, delivery mode, maternal history, blood pressure (BP), heart rate (HR), pulse rate (PR), respiratory rate (RR), body temperature (BT), and total input and output.

### Annotation process

In this study, IMV was defined as occurring in the following two situations based on patient data:

- Insertion or reinsertion of an endotracheal tube
- Use of a ventilator

Additionally, because the purpose of this model was to predict intubation 8 h in advance, events up to 8 h prior to the occurrence of intubation were labeled as events in the dataset. We directly annotated the intubation date and time based on the text-type nursing records. Referring to the nursing record, “Intubation was performed,” preprocessing was performed to determine what text meant the application of IMV.

### The proposed approach

#### Data preprocessing

**Fig. 2:**
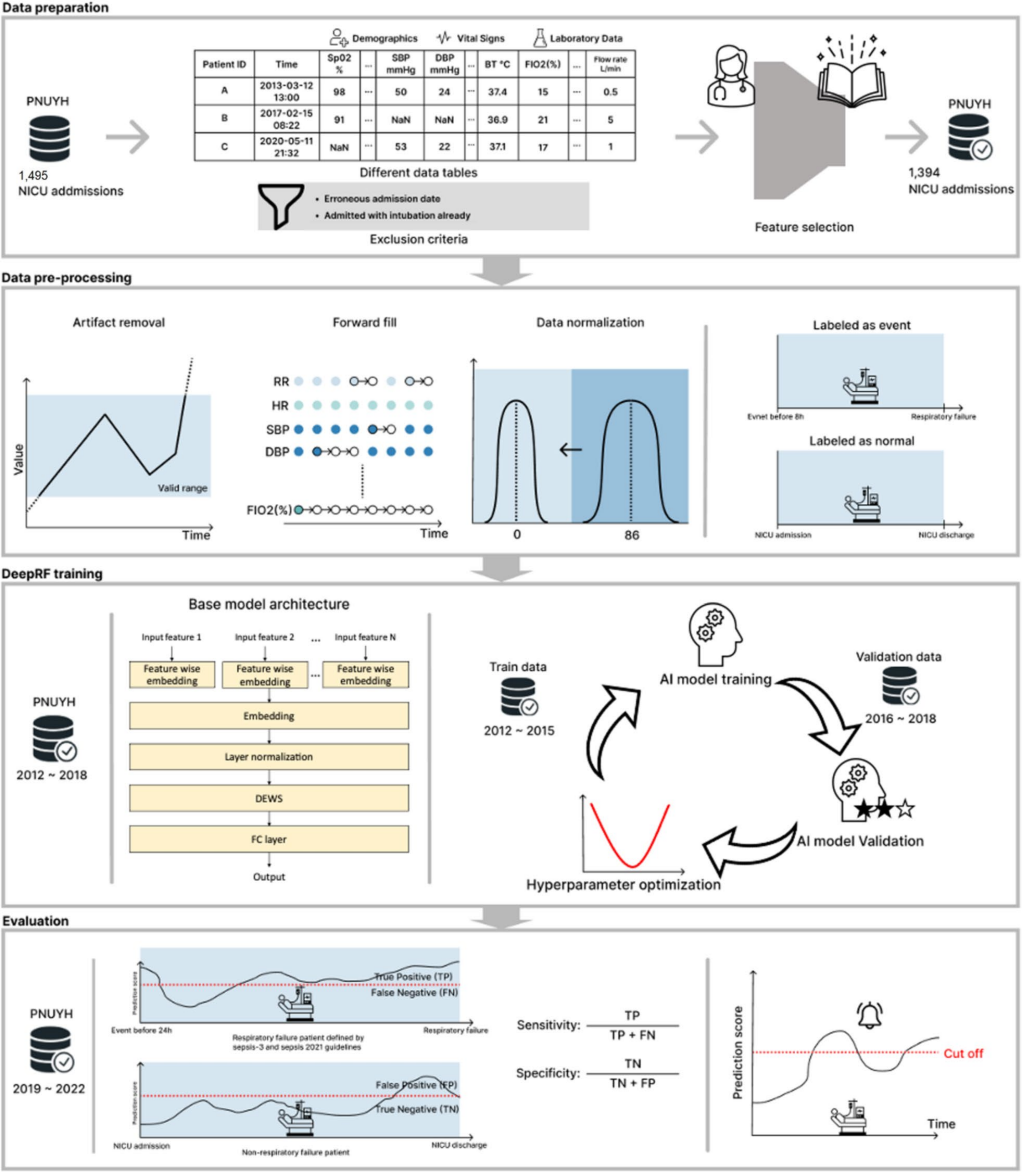
Overall methodology of data preparation, pre-processing, DeepRF, and evaluation in neonatal respiratory failure prediction model

Figure 2 illustrates several data preprocessing techniques, such as artifact removal, forward filling, and data normalization, used in the study. Experimental sciences utilize a theoretical model to represent real-world phenomena, and within particle physics, applying a “5 sigma” criterion is conventional when announcing a discovery. Therefore, any data points not included within the 5-sigma range were deemed anomalies and treated as artifacts, and were thus excluded from the analysis. EHR data often contain missing values that can interfere with the development of AI models. To address this issue, data imputation is commonly used and can effectively improve the quality of the data and enhance the performance of the model [20]. This study used the forward-fill method as the primary imputation method. If no previous data were available, the global median values of the features were inserted. Lastly, data normalization was performed for each feature.

#### Sequence windowing

Incorporating information from adjacent records can be beneficial and is commonly employed when training with EHR data and learning from records. Therefore, in this experiment, we used a window of 60 records for training. We also considered additional factors, such as the measurement time of each record, the time difference between consecutive measurements, and the variation in values between the previous measurement factors (Additional file 1: Fig. 1).

#### Data resampling

The class imbalance problem is widely recognized as a significant challenge when training AI models; our NICU data is no exception in this regard. The number of EHRs of 1,394 patients used in the model development was 216,490. Of these, EHRs for IMV accounted for only approximately 3.39% (7,329). We used a data resampling technique to address the problem [21]. Ideally, there would be a 1:1 ratio between event data and normal data. However, in such cases, the number of normal data instances involved in the training process might decrease, leading to potential trade-offs. In our optimization experiments, we introduced the normal-event ratio as a hyperparameter and compared the results to address this issue. The search space for the normal-event ratio was set to 1:1, 2:1, and 4:1. Upon evaluation, the 4:1 ratio demonstrated the best performance; thus, the 4:1 ratio was used.

### Model training and validation

#### Model development

The training, validation, and test datasets were set from March 4, 2012 to March 3, 2016, from March 4, 2016 to March 3, 2019, from March 4, 2019 to March 3, 2022, respectively. Considering the data distribution shift, we validated data from a time close to the test set rather than using cross-validation. The proposed model architecture is as follows (Additional file 1: Table 2). First, feature embedding based on feature-wise fully connected (FC) layers was performed, which was then input into three bidirectional LSTM layers [22]. After passing through the five FC layers, the final RF risk score was obtained using softmax. The detailed model architecture has been described in the Additional file 1: Table 2. Hyperparameter tuning was performed using the random-search method, with experiments conducted over 100 times [23]. For regularization, dropout was applied. The hyperparameter tuning results indicated that the optimal dropout ratio was 0.6 for the FC layer and 0.3 for the LSTM layer without regularizers. The AdamW optimizer was used during model training, and binary cross-entropy was used as the loss function [24, 25].

#### Comparison with existing methods

In this study, we compared the proposed model with the following methods: First, we used the newborn early warning score system (NEWS), which has been used in clinical settings, and an ML algorithm-based method that has been widely used because of its good performance [26, 27]. For the ML-based method, we used Random Forest for the decision tree series and XGBoost for the boosting series using the same input feature as the proposed model [28, 29]. Additionally, the definition of RF depends on the availability of PaO_2_ and FiO_2_. Continuous monitoring of pulse oximetry-derived hemoglobin oxygen saturation (SpO_2_) can be utilized in the clinical setting to estimate the present value of PaO_2_ [30, 31]. From this perspective, RF can be predicted solely by using SpO_2_ and FiO_2_. Therefore, we compared XGBoost using only two features: SpO_2_ and FiO_2_ [32].

#### Evaluation methods

To compare predictive performance, we used the area under the receiver operating characteristic (AUROC) and the area under the precision-recall curve (AUPRC) metrics and compared the sensitivity, positive predictive value (PPV), negative predictive value (NPV), positive likelihood ratio (LHR)+, and negative likelihood ratio (LHR-) at the same specificity as the NEWS value. To compare the alarm performance, we calculated the mean alarm count per day (MACPD) per 100 beds and calculated MACPD at the same sensitivity for all methods.

#### Software

EHR entries were extracted and pre-processed using the NumPy (version 1.20.3) and Pandas (version 1.5.2) libraries of the Python programming language, specifically version 3.8.13 (Python Software Foundation, Fredericksburg, VA, USA). Statistical analyses between groups were performed using the SciPy package version 1.10.0. Random Forest was implemented during model training using Scikit-learn (Scikit-learn Contributors, version 1.2.0). The XGB algorithm was applied using the XGBoost package (version 1.7.3). The evaluation was conducted using the Scikit-learn package along with the Shapley Additive exPlanations (SHAP) values (version 0.41.0).

## Results

### Baseline characteristics

A total of 1,394 neonatal patients were included in this study, with a mean GA of 36.61±3.25 weeks (Table 1). The mean birth weight and height were 2,734.01 ± 784.98 g and 46.93±4.33 cm, respectively. Intrauterine growth restriction (IUGR) in the 10th percentile was 12.3%, and that in the 3rd percentile was 5.6%. Vaginal delivery was performed in 67.0% of the patients, and 59.7% were male. The IMV group was more likely to have a lower GA, birth weight, and height and a higher clinical risk index for babies (CRIP II) score than the non-IMV group (*P* <0.05). The duration of hospitalization was notably longer in the IMV group (32.06±31.93 vs. 8.98±7.91 days) than in the non-IMV group. Respiratory distress syndrome (RDS), patent ductus arteriosus (PDA), bronchopulmonary dysplasia (BPD), premature retinopathy of prematurity (ROP), and necrotizing enterocolitis (NEC) were more frequently observed in the IMV group (*P*<0.05). The overall mortality rate was 3.1%.

**Table 1:** Comparison of the demographic data of neonatal patients

	**IMV patient** **(*n* = 505)**	**Non-IMV patient** **(*n* = 889)**	**All patient** **(*N* = 1,394)**	***P* value**
**Maternal characteristics**				
Maternal hypertension, n (%)	10 (2.0)	17 (1.9)	27 (1.9)	0.9299
GDM, n (%)	21 (4.2)	37 (4.2)	58 (4.2)	0.9974
Antenatal steroid, n (%)	0(0.0)	2(0.2)	2(0.1)	0.1574
Delivery mode				
Vaginal delivery, n (%)	341 (67.5)	593 (66.7)	934 (67.0)	0.7540
Cesarean section, n (%)	164 (32.48)	296 (33.3)	460 (33.0)	0.7540
**Patient characteristics**				
Gestational age (week)	35.16 ± 4.17	37.44 ± 2.18	36.61 ± 3.25	< 0.001
Birth weight (g)	2,446.33 ± 903.33	2,897.44 ± 655.50	2,734.01 ± 784.98	< 0.001
Birth height (cm)	45.49 ± 5.11	47.72 ± 3.60	46.93 ± 4.33	< 0.001
IUGR, n (%)	58 (11.5)	113 (12.7)	171 (12.3)	0.4978
< 3 percentile, n (%)	28 (5.5)	50 (5.6)	78 (5.6)	0.9503
Male, n (%)	314 (62.2)	518 (58.3)	832 (59.7)	0.1510
CRIB II score	1.79 ± 2.18	1.43 ± 1.50	1.43 ± 1.50	< 0.001
Surfactant administration, n (%)	0 (0.0)	89 (10.0)	89 (6.4)	< 0.001
Underlying comorbidities, n (%)				
RDS	260 (51.5)	4(0.5)	264 (18.9)	< 0.001
PDA	90 (17.9)	20 (2.2)	110 (7.9)	< 0.001
BPD	21 (4.2)	2 (0.2)	23 (1.7)	< 0.001
ROP	7 (1.4)	2 (0.2)	9 (0.7)	0.0334
NEC	16 (3.2)	3 (0.3)	19 (1.4)	< 0.001
Duration of hospitalization, mean ± SD (day)	32.06 ± 31.93	8.98 ± 7.91	17.34 ± 23.06	< 0.001
Mortality, n (%)	38 (7.5)	5 (0.6)	43 (3.1)	0.0000

*Abbreviations*: *BPD* Bronchopulmonary dysplasia, *CRIP* Clinical Risk Index for Babies, *GDM* Gestational diabetes mellitus, *NEC* Necrotizing enterocolitis, *IMV* Invasive mechanical ventilation, *IUGR* Intrauterine growth restriction, *RDS* Respiratory distress syndrome, *ROP* Retinopathy of prematurity, *SD* Standard deviation, *PDA* Patent ductus arteriosus

#### Predictive performance

**Fig. 3:**
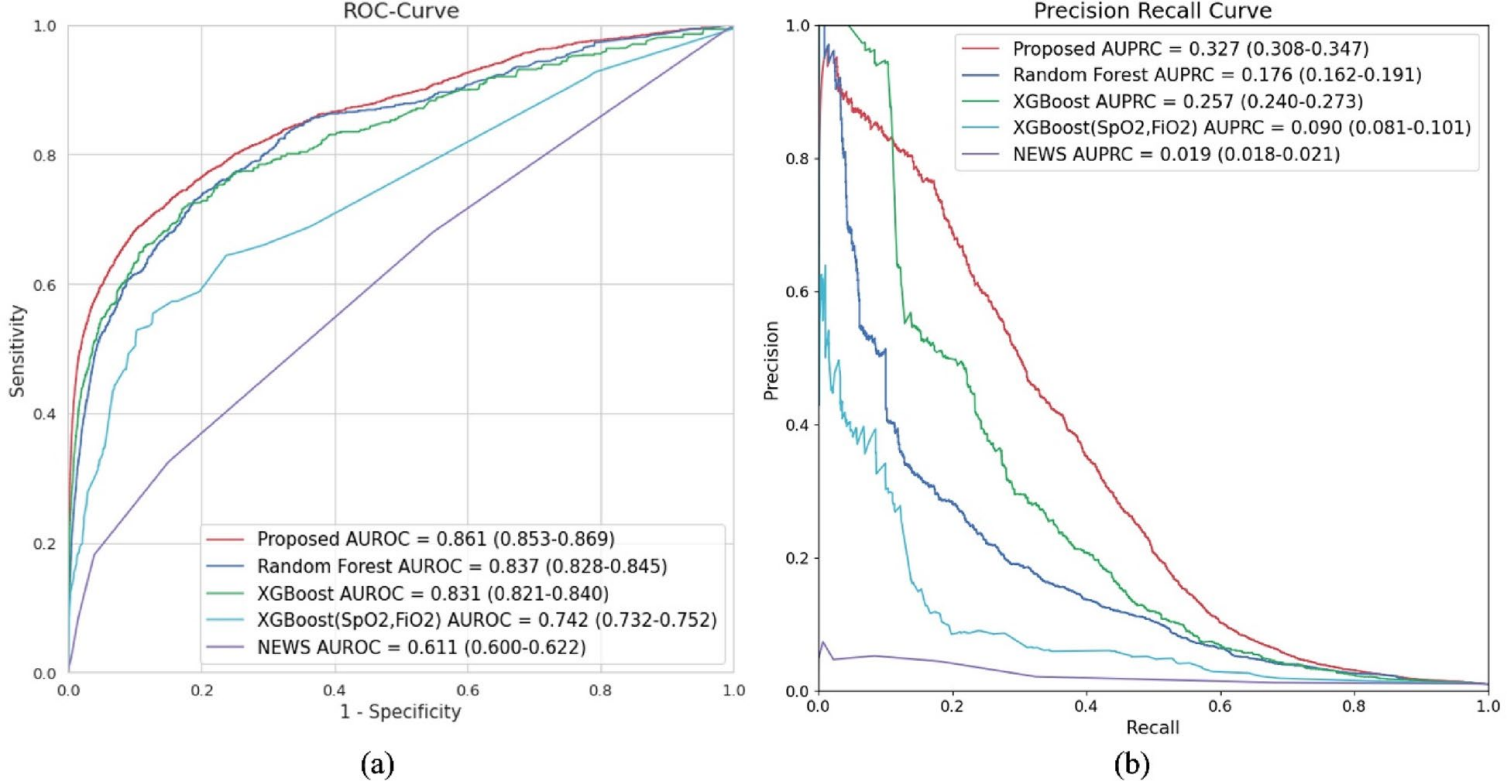
Predictive performance based on the proposed method, Random Forest, XGBoost, XGBoost (SpO2, FiO2), and NEWS. **a** Receiver operating characteristic (ROC) curves, **b** Precision-recall curves. Abbreviations: AUROC, area under the receiver operating characteristic; AUPRC, area under the precision-recall curve; NEWS, Newborn early warning score system; ROC, receiver operating characteristic; XGBoost, extreme gradient boosting

Our first experiment compared the predictive performances of various approaches. Fig. 3a shows the receiver operating characteristic (ROC) curves for different models. The proposed model achieved the highest AUROC (0.861) compared with the other models. Random Forest and XGBoost had similar performances, with AUROCs of 0.837 and 0.831, respectively. The XGBoost model, which utilized only two features (SpO_2_ and FiO_2_), also demonstrated an AUROC of 0.742. Although this value was lower than the AUROC achieved by the model incorporating all the selected features, it outperformed NEWS (AUROC: 0.611). In Fig. 3b, the precision-recall curves are compared. The developed model exhibited the highest AUPRC (0.327). Unlike the AUROC results, XGBoost had a better AUPRC than Random Forest (0.257 vs. 0.176). The XGBoost model, which utilized only two features, had a lower performance (AUPRC: 0.090) than the model that incorporated all selected features; it outperformed NEWS (AUPRC: 0.019).

Significant differences were observed in the terms of GA and birth weight between the IMV and non-IMV groups (Table 1). Based on these factors, we performed a subgroup analysis. In Additional file 1: Fig. 2 (a), We compared the AUROC of all models for each group according to the GA. The proposed model performed better for all groups and provided more accurate predictions as GA decreased. However, the proposed model and Random Forest showed similar performances in the group with a gestation period of 35 weeks. Additionally, as shown in Additional file 1: Fig. 2 (b), we analyzed group-specific AUROC according to birth weight. In this case, the proposed model also showed the highest performance, and we found that it had a strong performance regardless of the group. The proposed model showed similar performance to Random Forest and XGBoost in the group with birth weight ≤1.75 kg and ≥4.25 kg, respectively. However, the p-values are 0.295 and 0.014, respectively, indicating that the results are not significant.

In Table 2, of the 5 parameters in the widely used NEWS, comparisons were made using 2, 3, 4, and 5 or more parameters to assess the performance of all models corresponding to the same specificity. The proposed model stands out as it achieved the highest sensitivity, LHR+, and the lowest LHR-. Specificity relates to the number of alarms; when the number of alarms is consistent, the proposed model delivers the best performance.

**Table 2:** Comparison of performance for invasive mechanical ventilation prediction models in NICU with a consistent specificity threshold

Models* (*≥ *threshold)*	Specificity	Sensitivity	PPV	NPV	LHR +	LHR-
NEWS ≥ 2	0.8492	0.3248	0.0209	0.9921	2.1545	0.7950
XGBoost(SpO2,FiO2) *(*≥ *0.3383)*	0.8535	0.5150	0.0336	0.9944	3.5165	0.5681
XGBoost *(*≥ *0.2487)*	0.8492	0.6845	0.0430	0.9963	4.5401	0.3714
Random forest *(*≥ *0.4018)*	0.8492	0.6774	0.0426	0.9962	4.4948	0.3797
Proposed *(*≥ *0.5207)*	0.8493	0.7250	0.0455	0.9968	4.8116	0.3237
NEWS ≥ 3	0.9608	0.1812	0.0438	0.9916	4.6334	0.8520
XGBoost(SpO2,FiO2) *(*≥ *0.5187)*	0.9552	0.3121	0.0646	0.9929	6.9723	0.7200
XGBoost *(*≥ *0.4932)*	0.9608	0.5108	0.1145	0.9949	13.0595	0.5090
Random forest *(*≥ *0.4960)*	0.9608	0.4868	0.1097	0.9947	12.4397	0.5340
Proposed *(*≥ *0.6554)*	0.9608	0.5739	0.1268	0.9956	14.6601	0.4434
NEWS ≥ 4	0.9848	0.0838	0.0520	0.9908	5.5429	0.9302
XGBoost(SpO2,FiO2) *(*≥ *0.6668)*	0.9845	0.1440	0.0843	0.9914	9.3021	0.8693
XGBoost *(*≥ *0.6669)*	0.9848	0.3992	0.2067	0.9939	26.3014	0.6100
Random forest *(*≥ *0.5283)*	0.9848	0.3248	0.1749	0.9932	21.3943	0.6855
Proposed *(*≥ *0.7303)*	0.9848	0.4825	0.2403	0.9948	31.9286	0.5253
NEWS ≥ 5	0.9954	0.0225	0.0467	0.9903	4.9483	0.9818
XGBoost(SpO2,FiO2) *(*≥ *0.7207)*	0.9931	0.0880	0.1131	0.9909	12.8826	0.9182
XGBoost *(*≥ *0.7887)*	0.9954	0.2622	0.3638	0.9927	57.7161	0.7411
Random forest *(*≥ *0.5829)*	0.9954	0.1836	0.2869	0.9919	40.6202	0.8200
Proposed *(*≥ *0.8065)*	0.9954	0.3455	0.4289	0.9935	75.8233	0.6574

*Abbreviations*: *LHR* Likelihood ratio, *NEWS* Newborn early warning score system, *NICU* Neonatal intensive care unit, *NPV* Negative predictive value, *PPV* Positive predictive value, *XGBoost* Extreme gradient boosting

#### Alarming performance

**Fig. 4:**
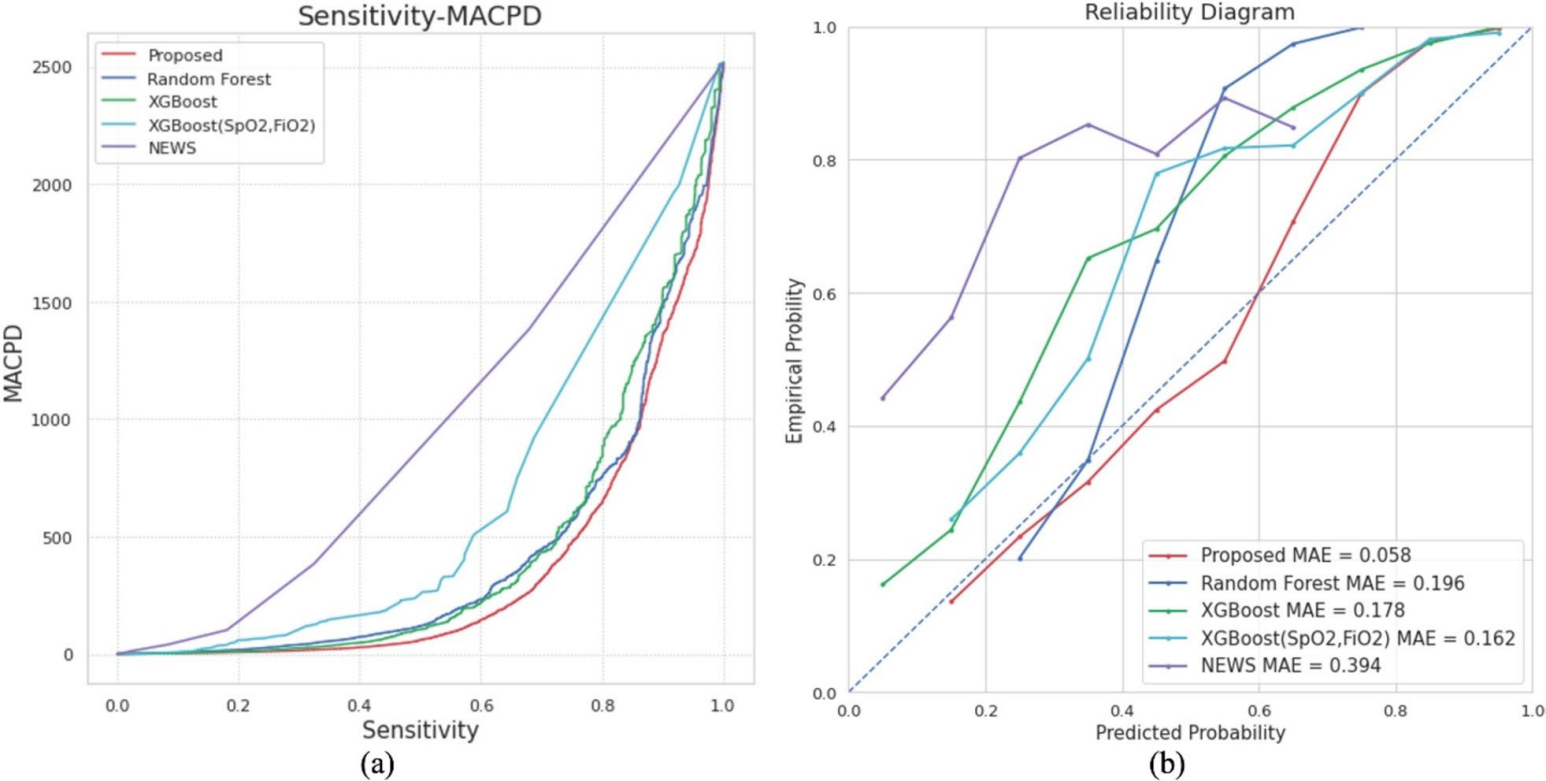
Alarming performance and Reliability diagram. **a** Comparison of the mean alarm count per day per 100 beds at the same sensitivity point for predicting respiratory failure in NICU. MACPD indicates the mean alarm count per day per 100 beds, and NEWS indicates the newborn early warning score. **b** Comparison of the calibration level for each model based on the reliability diagram. Abbreviations: MAE, mean absolute error; NEWS, Newborn Early Warning Score System; XGBoost, extreme gradient boosting

We compared MACPD using the same sensitivity level for all methods. From Fig. 4a and Table 3, it can be observed that the proposed method has the lowest alarm rate compared to all other methods at the same sensitivity level. This result indicates that the proposed method can detect the same number of high-risk patients with fewer alarms, significantly reducing the burden on the medical staff. In addition, the calibration level of the model is crucial when setting a threshold for the alarms for each model. We analyzed the reliability, as shown in Fig. 4b, and found that the proposed method had the best calibration level.

**Table 3:** Comparison of MACPD per 100 beds according to same sensitivity

Models* (*≥ *threshold)*	Sensitivity	MACPD
NEWS ≥ 1	0.6798	1382
XGBoost(SpO2,FiO2) *(*≥ *0.2145)*	0.6614	757
XGBoost *(*≥ *0.2569)*	0.6798	374
Random forest *(*≥ *0.3943)*	0.6798	406
Proposed *(*≥ *0.5588)*	0.6798	226
NEWS ≥ 2	0.3248	383
XGBoost(SpO2,FiO2) *(*≥ *0.4885)*	0.3276	125
XGBoost *(*≥ *0.7409)*	0.3243	28
Random forest *(*≥ *0.5283)*	0.3248	45
Proposed *(*≥ *0.8447)*	0.3248	14
NEWS ≥ 3	0.1812	101
XGBoost(SpO2,FiO2) *(*≥ *0.6669)*	0.1817	38
XGBoost *(*≥ *0.8494)*	0.1826	8
Random forest *(*≥ *0.5836)*	0.1812	15
Proposed *(*≥ *0.9126)*	0.1812	6
NEWS ≥ 4	0.0838	39
XGBoost(SpO2,FiO2) *(*≥ *0.8099)*	0.0828	5
XGBoost *(*≥ *0.9132)*	0.0819	2
Random forest *(*≥ *0.6340)*	0.0838	3
Proposed *(*≥ *0.9520)*	0.0838	2

*Abbreviations*: *MACPD* Mean alarm count per day, *NEWS* Newborn early warning score system, *NICU* Neonatal intensive care unit, *XGBoost* Extreme gradient boosting

#### Inspection of model features

**Fig. 5:**
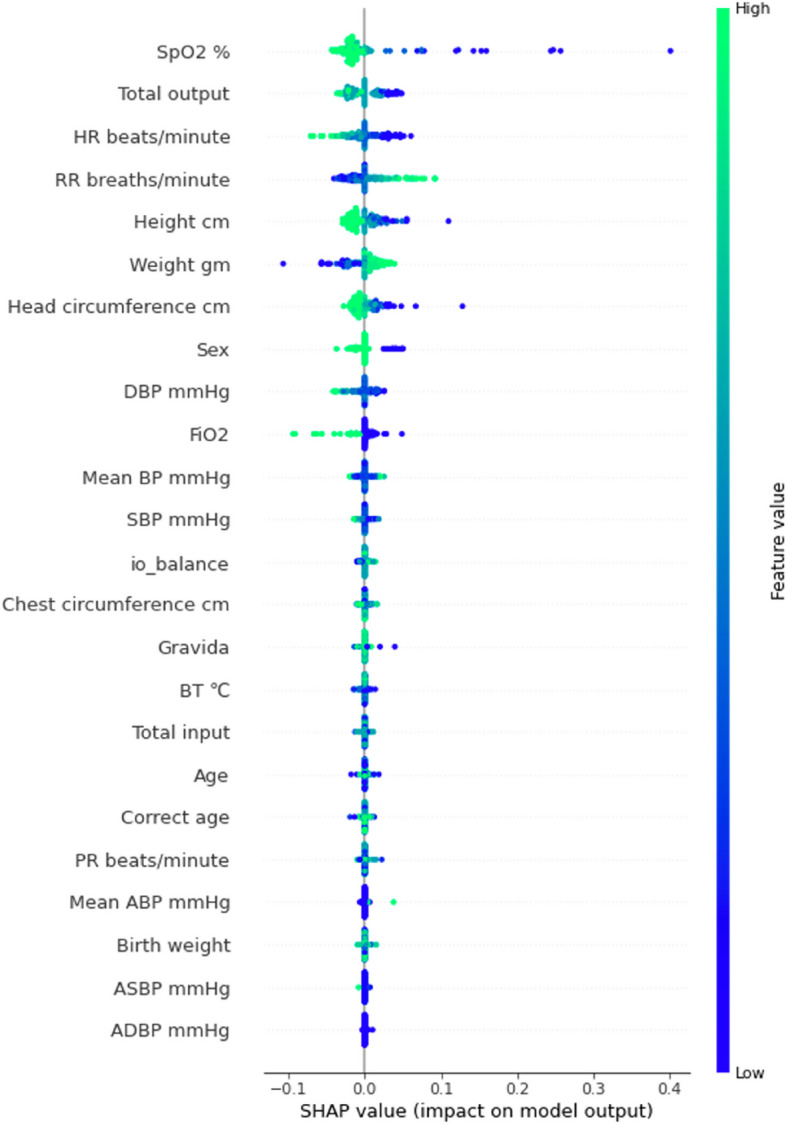
Feature importance according to SHapley Additive explanation (SHAP) value. Abbreviations: ABP, arterial blood pressure; BT, body temperature; DBP, diastolic blood pressure; HR, heart rate; IO, input and output; PR, pulse rate; SBP, systolic blood pressure

The overall importance of the predictor variables of the proposed model showed SpO_2_ as the most important feature, and the second most important feature was the total output, including urine and feces (Fig. 5). Heart and respiratory rates per minute were the third- and fourth-most important features, respectively.

## Discussion

In this study, we demonstrated DL to support clinical decision-making concerning applying IMV among neonates using non-invasive methods such as monitoring vital signs and demographic information. RF is a critical condition commonly observed in newborns admitted to the NICU, leading to an increased mortality rate [33]. Repeated or prolonged episodes of desaturation and tachypnea, including hypoxia, neurodevelopmental impairment, persistent pulmonary hypertension, and cardiac arrest, may worsen the prognosis. The rapid and accurate decision of intubation is vital to increase survival [1, 32].

Among the articles published to date, an accurate tool for predicting intubation has yet to be established. Several neonatal severity scoring systems have been developed to predict the prognosis of critically ill neonates, including the Clinical Risk Index for Babies II (CRIB II), Neonatal Therapeutic Intervention Scoring System (NTISS), Score for Neonatal Acute Physiology II (SNAP II), Score for Neonatal Acute Physiology with Perinatal Extension II (SNAPPE-II), and Modified Sick Neonatal Score (MSNS). These scores accurately predicted mortality in the NICU, and the AUCs were approximately 0.86–0.91 [34, 35]. However, these scores were originally designed to assess the worst clinical status found in the first 24 h after admission [36]. The proposed model achieved the highest predictive accuracy for respiratory deterioration requiring IMV. Both Random Forest and XGBoost exhibited similar performances. The XGBoost model that utilized only two features (SpO_2_ and FiO_2_) had a lower AUROC compare to the model that incorporated all selected features. We also found that the proposed model performed better for all groups and tended to make more accurate predictions for lower GA.

We also compared MACPD using the same sensitivity level for all methods. Poncette et al. [37] described that in one of the most digitized hospitals with an increasing number of novel medical devices with their own alarms, the sheer number of alarms frequently overwhelms clinicians. Kierra Jones [38] documented that Johns Hopkins reported an average of 350 alerts per bed per day, and one intensive care unit’s (ICU) average was 771 daily. This can cause alarm fatigue, and caregivers are more likely to ignore or have trouble distinguishing between the alarms. In this study, the proposed method had the lowest alarm rate compared to all other methods at the same sensitivity level. This result indicates that the proposed method can detect the same number of high-risk patients with fewer alarms, which can help reduce alarm fatigue and workload. It also improves the selection of alarms requiring immediate intervention, provides earlier recognition of treatment, and directs care toward more efficient and individualized situations.

The strengths of our study were two-fold. First, there were no restrictions on the equipment or human resources required to use the proposed model. We developed a model that makes accurate predictions with minimal key features: GA, birth weight, corrected age, gravida, head circumference, body weight, height, chest circumference at birth, sex, FiO_2_, SpO_2_, BT, systolic, diastolic, and mean BP, HR, PR, and RR, which can be obtained non-invasively. The model is versatile and can be used in primary to tertiary hospitals, even in situations with limited laboratory equipment or a shortage of specialists. If the risk of IMV application in a primary hospital is high, a transfer to a tertiary hospital can be promptly considered. Secondly, the proposed model is valuable for determining whether IMV is necessary for a patient hospitalized for several hours or days. Immediately after birth, the need for IMV support becomes conspicuously evident within the framework of the neonatal resuscitation program. This encompasses indicators such as apnea, gasping, desaturation, and bradycardia. Attention may wane several hours or days into hospitalization, even though close monitoring and accurate judgment by medical staff remain necessary throughout the hospitalization period. By developing the proposed model, intubation and mechanical ventilation support can be initiated without delay due to early detection with a reduced alarm burden.

The current study has some limitations that should be addressed in future studies. First, it was limited to a single hospital, which could have affected the generalizability of the model. This is because clinicians use different criteria to determine the necessity for intubation. The application of the proposed model requires further external validation in other institutions, and a bias in therapeutic strategies is inevitable. Second, patients who were intubated before admission were excluded, and most of them were extremely low birth weight infants (ELBWI). Data pertaining to the application of IMV in cases of extreme immaturity are crucial. The ELBWI exhibited insufficient self-respiration and decreased physical activity immediately after birth. Moreover, ELBWI generally received prophylactic surfactants via an endotracheal tube. In the future, we aim to monitor and evaluate each patient from the delivery room to the NICU. Third, outborn patients did not have sufficient information regarding their maternal history, such as prenatal ultrasound or laboratory test results, which are critical factors affecting neonatal lung disease. This prospective study aimed to collect various types of maternal data.

## Conclusion

Using non-invasive data, we demonstrated the performance of a DL-based approach in predicting the need for mechanical ventilation in neonates in the NICU. The results of the predictive and alarm performances were superior for the proposed model compared to the other models. DL approaches offer an accurate and standardized way to predict applying IMV in neonatal patients, enabling advanced bedside neonatal care and the utilization of more sophisticated techniques.

### Supplementary Information

**Additional file 1: Table 1**. The percentage of missing data. **Table 2.** The proposed model architecture. hidden_dim=32, input_dim=83. **Table 3.** Significance testing of proposed model and existing models.** Fig. 1.** Sequence windowing. Yellow box means current EHR data and blue box means sequence of recodes to support model training and prediction when sequence length is L. Red box is target label according to sequence window. **Fig. 2.** Sub-group analysis. (a) Area under the curve (AUC) according to gestational age and (b) AUC according to birth weight.

## Data Availability

The datasets supporting the conclusions of this article are included within the article and its additional files. Further inquiries can be directed to the corresponding author.

## Abbreviations

AI: Artificial intelligence
ANN: Artificial neural networks
AUPRC: Area under the precision-recall curve
AUROC: Area under the receiver operating characteristic
BP: Blood pressure
BPD: Bronchopulmonary dysplasia
BT: Body temperature
CNN: Convolutional neural networks
DL: Deep learning
EHR: Electronic health records
ELBWI: Extreme low birthweight infant
FC: Fully connected
GA: Gestational age
GDM: Gestational diabetes mellitus
HR: Heart rate
ICU: Intensive care unit
IMV: Invasive mechanical ventilation
IUGR: Intrauterine growth restriction
LDA: Linear discriminant analysis
LHR: Likelihood ratio
LSTM: Long short-term models
MACPD: Mean alarm count per day
MAE: Mean absolute error
ML: Machine learning
MSNS: Modified Sick Neonatal Score
NEC: Necrotizing enterocolitis
NEWS: Newborn early warning score system
NICU: Neonatal intensive care unit
NPV: Negative predictive value
NTISS: Neonatal Therapeutic Intervention Scoring System
PDA: Patent ductus arteriosus
PPV: Positive predictive value
PR: Pulse rate
RDS: Respiratory distress syndrome
RF: Respiratory failure
ROC: Receiver operating characteristic
ROP: Retinopathy of prematurity
RR: Respiratory rate
SHAP: Shapley Additive exPlanations
SNAP: Score for Neonatal Acute Physiology
SNAPPE: Score for Neonatal Acute Physiology with Perinatal Extension
SVM: Support vector machine
XGBoost: Extreme gradient boosting
