# Supplementary material for: Early prediction of need for invasive mechanical ventilation in the neonatal intensive care unit using artificial intelligence and electronic health records: a clinical study
Source: BMC Pediatr. 2023 Oct 23;23:525. doi: 10.1186/s12887-023-04350-1 (PMC10591351; doi:10.1186/s12887-023-04350-1)
Supplement: Supplementary file 1 — Additional file 1: Table 1. The percentage of missing data. Table 2. The proposed model architecture. hidden_dim=32, input_dim=83. Table 3. Significance testing of proposed model and existing models. Fig. 1. Sequence windowing. Yellow box means current EHR data and blue box means sequence of recodes to support model training and prediction when sequence length is L. Red box is target label according to sequence window. Fig. 2. Sub-group analysis. (a) Area under the curve (AUC) according to gestational age and (b) AUC according to birth weight. [file 12887_2023_4350_MOESM1_ESM.docx]

***Additional file 1***

Additional file 1, Table 1. The percentage of missing data

| **#** | **Name** | **Missing rate (%)** |
| --- | --- | --- |
| 1 | Age | 0.00 |
| 2 | Birth weight | 0.02 |
| 3 | Corrected age | 0.31 |
| 4 | Gravida | 0.16 |
| 5 | Head circumference | 98.90 |
| 6 | Weight | 95.41 |
| 7 | Height | 98.90 |
| 8 | Chest circumference at birth | 99.23 |
| 9 | Sex | 0.00 |
| 10 | FiO_2_ | 98.90 |
| 11 | SpO_2_ | 5.81 |
| 12 | Body temperature | 76.56 |
| 13 | Diastolic Blood pressure, NIBP | 80.24 |
| 14 | Heart rate | 5.41 |
| 15 | Mean ABP | 98.93 |
| 16 | Pulse rate | 10.20 |
| 17 | Respiratory rate | 5.37 |
| 18 | Systolic blood pressure, NIBP | 80.24 |
| 19 | Flow rate | 95.36 |
| 20 | Input-output balance | 95.67 |
| 21 | Total input | 95.66 |
| 22 | Total output | 95.67 |
| 23 | Systolic pressure, ABP | 98.93 |
| 24 | Diastolic pressure, ABP | 98.93 |
| 25 | Mean NIBP | 80.24 |

Abbreviations: ABP, arterial blood pressure; NIBP, noninvasive blood pressure

Additional file 1, Table 2. The proposed model architecture. hidden_dim=32, input_dim=83.

| **Layer name** | **Components** |
| --- | --- |
| Feature wise embedding | Linear1(in_features=1, out_features=8, bias=True)  Linear2(in_features=8, out_features=16, bias=True)  Dropout(0.4) |
| Embedding | Linear3(in_features=input_dim*16, out_features=hidden_dim, bias=True) |
| DEWS | LSTM1(in_features=hidden_dim, out_features=hidden_dim, bias=True)  LSTM2(in_features=input_dim+hidden_dim*2, out_features=hidden_dim, bias=True)  LSTM3(in_features=input_dim+hidden_dim*4, out_features=hidden_dim, bias=True)  Dropout(0.3) |
| FC layer | Fully-connected layers followed by a softmax function  Dropout(0.6) |

Additional file 1, Table 3. Significance testing of proposed model and existing models

| **AUROC** | | | |
| --- | --- | --- | --- |
| **Target model** | | ***P* value** | |
| Proposed | Random Forest | 4.720048e-49 | <0.05 |
|  | XGBoost | 4.212387e-59 | <0.05 |
|  | XGBoost(SpO2, FiO2) | 1.677730e-149 | <0.05 |
|  | NEWS | 8.935458e-217 | <0.05 |
| **AUPRC** | | | |
| **Target model** | | ***P* value** | |
| Proposed | Random Forest | 1.617579e-130 | <0.05 |
|  | XGBoost | 7.696515e-70 | <0.05 |
|  | XGBoost(SpO2, FiO2) | 6.297270e-175 | <0.05 |
|  | NEWS | 1.378902e-208 | <0.05 |


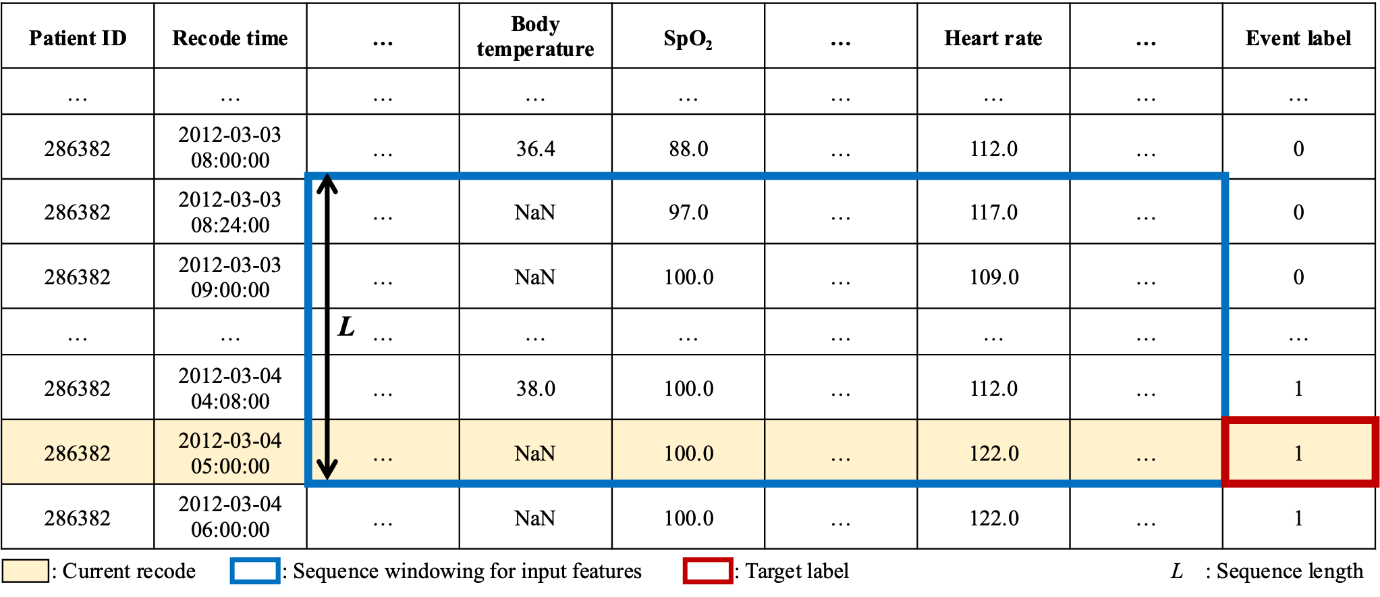
 Additional file 1, Fig. 1. Sequence windowing. Yellow box means current EHR data and blue box means sequence of recodes to support model training and prediction when sequence length is L. Red box is target label according to sequence window.


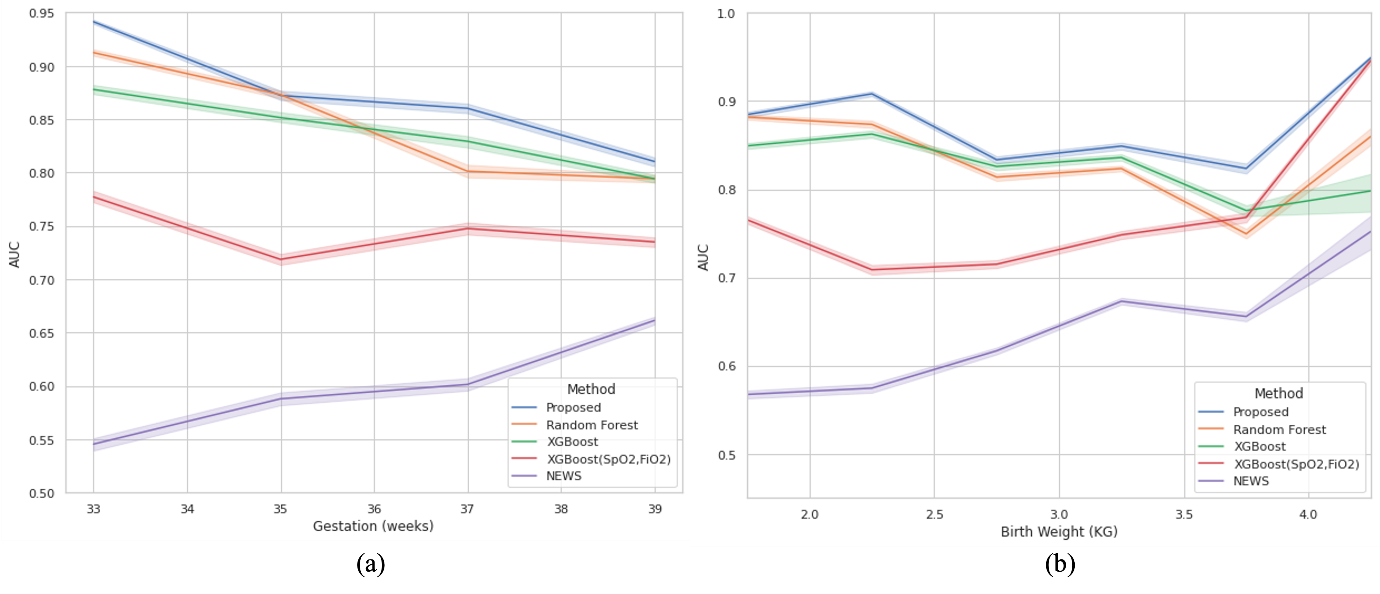


Additional file 1, Fig. 2. Sub-group analysis. (a) Area under the curve (AUC) according to gestational age and (b) AUC according to birth weight.
